# You are what you eat: diet shapes body composition, personality and behavioural stability

**DOI:** 10.1186/s12862-016-0852-4

**Published:** 2017-01-10

**Authors:** Chang S. Han, Niels J. Dingemanse

**Affiliations:** 1 Behavioural Ecology, Department of Biology, Ludwig-Maximilians University of Munich, Großhaderner Str. 2, 82152 Planegg-Martinsried, Germany; 2 Current address: School of Biological Sciences, University of Queensland, St Lucia, 4072 Australia

**Keywords:** Behavioural stability, Developmental plasticity, Diet, Personality, Repeatability, Heterogeneous residual within-individual variance

## Abstract

**Background:**

Behavioural phenotypes vary within and among individuals. While early-life experiences have repeatedly been proposed to underpin interactions between these two hierarchical levels, the environmental factors causing such effects remain under-studied. We tested whether an individual’s diet affected both its body composition, average behaviour (thereby causing among-individual variation or ‘personality’) and within-individual variability in behaviour and body weight (thereby causing among-individual differences in residual within-individual variance or ‘stability’), using the Southern field cricket *Gryllus bimaculatus* as a model. We further asked whether effects of diet on the expression of these variance components were sex-specific.

**Methods:**

Manipulating both juvenile and adult diet in a full factorial design, individuals were put, in each life-stage, on a diet that was either relatively high in carbohydrates or relatively high in protein. We subsequently measured the expression of multiple behavioural (exploration, aggression and mating activity) and morphological traits (body weight and lipid mass) during adulthood.

**Results:**

Dietary history affected both average phenotype and level of within-individual variability: males raised as juveniles on high-protein diets were heavier, more aggressive, more active during mating, and behaviourally less stable, than conspecifics raised on high-carbohydrate diets. Females preferred more protein in their diet compared to males, and dietary history affected average phenotype and within-individual variability in a sex-specific manner: individuals raised on high-protein diets were behaviourally less stable in their aggressiveness but this effect was only present in males. Diet also influenced individual differences in male body weight, but within-individual variance in female body weight.

**Discussion:**

This study thereby provides experimental evidence that dietary history explains both heterogeneous residual within-individual variance (i.e., individual variation in ‘behavioural stability’) and individual differences in average behaviour (i.e., ‘personality’), though dietary effects were notably trait-specific. These findings call for future studies integrating proximate and ultimate perspectives on the role of diet in the evolution of repeatedly expressed traits, such as behaviour and body weight.

**Electronic supplementary material:**

The online version of this article (doi:10.1186/s12862-016-0852-4) contains supplementary material, which is available to authorized users.

## Background

Behavioural ecologists increasingly focus on why individuals differ in average behaviour (i.e., why there is among-individual variation or ‘animal personality’) and behavioural plasticity (i.e., why within-individual variation or ‘behavioural stability’ differs among individuals) [1–3]. Quantitative genetics studies imply that, on average, 50% of this individual variation in behaviour is due to additive genetic effects (reviewed in [4]). Environmental factors that permanently affect the phenotype therefore likely play an equally important role in shaping individual behaviour. Indeed, various studies have experimentally demonstrated the importance of the early-life environment in permanently shaping behavioural phenotypes [5–9]. For example, studies on birds imply that food availability during early-life can shape both aggressiveness and exploratory tendency in adulthood [10].

Empirical studies increasingly quantify the contribution of among- and within-individual variation in shaping phenotypic variation observed in animal behaviour [3, 11]. Recent research indicates that the (relative) magnitudes of these two variance components can show sex-specificity and spatiotemporal variation within a single species [12–17]. This is in line with quantitative genetics studies showing that the expression of genetic variance is often a function of the environment [18]. For example, the additive genetic variance may either increase, or decrease, under more favourable (or less ‘stressful’) conditions [19, 20]. Behavioural examples are relatively few but include work on the house cricket (*Acheta domesticus*) showing that low quality diet increases within-individual stability in anti-predatory behaviour, and consequently increases its repeatability [16]. Similarly, adults of the western trilling crickets (*Gryllus integer*) exposed to bacterial pathogens during ontogeny are less repeatable (due to decreased among-individual variance) in boldness compared to controls [15]. Examples of environment-specific within-individual variation, furthermore, include work on red-winged blackbirds (*Agelaius phoeniceus*), where females decrease their within-individual variance in nestling provisioning effort with increasing nestling age [17], studies on hermit crabs (*Pagurus bernhardus*), where individuals increase their level of within-individual behavioural variability when faced with increased perceived predation risk [13], and studies on zebra finches (*Taeniopygia guttata*), where early dietary restriction decreases the amount of within-individual variance in general activity [14].

**Fig. 1:**
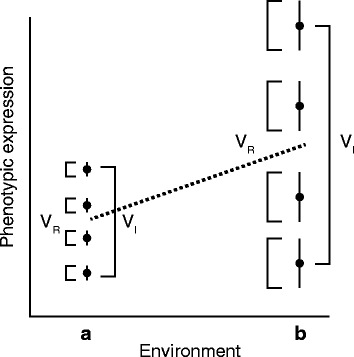
Schematic representation of how mean level, and within- and among individual variances were expected to differ between environments. Each dot with vertical line represents one theoretical individual. The black line represents the extent of variation in behaviour that is observed across observations of a single individual (i.e., within-individual variance, V_R_). The change in the length of black lines indicates the change in within-individual variance (V_R_) across environments. The variation among dots represents the variation among individuals in average phenotype (i.e., among-individual variance, V_I_). The dashed line indicates a change in population-level mean

Here we propose that the nutritional environment represents a key environmental factor determining the development and expression of behaviour, thus shaping personality and level of behavioural stability [21]. We further propose that there are three components of behaviour that can be affected by nutritional history. First, average level of behaviour may vary among individuals with different nutritional histories (represented by the dotted line in Fig. 1). Indeed, previous studies have demonstrated that poor nutrition can impair behavioural development and provide reliable cues to the expression of later behaviour [14, 22–28].

Second, the extent of individual differentiation (i.e., the amount of among-individual variance; V_I_) may differ among groups of individuals differing in nutritional history (variance component V_I_ in Fig. 1). For example, under high-quality dietary conditions (characterized by abundant resources or balanced nutrients), multiple strategies with equal fitness payoffs may exist by which resources are allocated among costly behaviours, while this might not be the case under low-quality conditions. If so, one would expect the among-individual variance in behaviour to be increased under high-quality dietary conditions [21] (Fig. 1). One may, by contrast, also expect the opposite pattern, for example, when low-quality dietary environments represent relatively novel environmental conditions where additional (‘cryptic’) genetic variance is expressed [29], leading to decreased among-individual variance under high-quality dietary conditions (e.g., [14, 30, 31]).

Third, behavioural instability (i.e., the amount of residual within-individual variance; V_R_) may also differ between individuals subjected to different nutritional histories (variance component V_R_ in Fig. 1). Owing to the costs associated with phenotypic plasticity [32], for example, only individuals experiencing high-quality nutritional environments might be able to invest in the sensory and processing apparatus necessary for expressing adaptive phenotypic plasticity. High-quality nutritional environments during development might therefore enable individuals to more flexibly respond to micro-environmental variation experienced during adulthood. For example, individuals subjected to high-quality nutritional environments during development might develop the capabilities to adjust their level of aggressiveness in response to changes in their social environment, whereas individuals with a low-quality dietary past might remain less flexible [33, 34]. This multitude of possible effects on behavioural variance components may thereby result in behavioural repeatabilities that vary between treatment groups [14, 28]. Importantly, as variances often increase as a function of the mean [35], effects of diet on mean behavioural phenotype are likely coupled with those on variance components (as illustrated in Fig. 1).

We further predict that effects of diet should differ between sexes and life stages. Sex-differences in sensitivity towards nutrient deficiency are expected because males and females often differ in their nutritional preference [26, 36–39]. In addition, as nutritional requirements typically change throughout an individual’s life time [40], diets experienced during early vs. late life are predicted to additively or interactively affect the expression of phenotypes in adulthood (e.g. [41–44]). In the butterfly *Harmonia axyridis*, for example, females raised on a poor diet during adulthood showed decreased fecundity only when not having experienced a poor diet as a larvae [42].

Here, we assessed how nutritional environments experienced during juvenile and adult life affected various behaviours and their variance components in males and females of the Southern field cricket (*G. bimaculatus*). Field crickets are well suited for testing the role of the nutritional environment in shaping personality and plasticity. Previous research on crickets has, for example, shown that the carbohydrate:protein (C:P) ratio of a diet affects the expression of morphology and reproductive behaviours [22, 25, 26, 44–46]. This is not surprising as protein is required for somatic development of nymphs, and eggs produced by females. Similarly, carbohydrate is needed to fuel general activity and male courtship behaviour. In our experiments, we therefore manipulated diets. We used a two-way factorial design with two juvenile diets (high-carbohydrate versus high-protein) and two adult diets (high-carbohydrate versus high-protein), and measured the expression of multiple behavioural (exploration, aggression and mating activity) and morphological (body weight and lipid mass) traits. Given the documented strong effects of the carbohydrate:protein (C:P) ratios on various key phenotypic traits [22, 25, 26, 44–46], dietary environments are generally predicted to alter body weight and the expression of behaviour, though we appreciate that such effects may also interact with social and non-social environmental factors, such as the amount of competition for resources or mates. Moreover, effects of diet on the expression of variance components (i.e., among- or within-individual variances) may also affect behavioural repeatability. We thus measured 1) nutritional preferences of juveniles, adult males and adult females and 2) nutritional intakes of juveniles and adults faced with imbalanced diets (namely, high-carbohydrate diets vs. high-protein diets, detailed below) (experiment 1). We then assessed whether 3) population-level mean trait values, 4) variance components and 5) repeatabilities differed across nutritional environments (experiment 2). We also tested 6) whether the effects of diet (on mean and variance components) differed across the sexes.

## Methods

### Cricket maintenance and diet preparation

For our experiments, we used the third generation of offspring of adult crickets collected from a natural population in Tuscany (Italy) in July 2013. By not using offspring of wild-caught adults we thereby avoided biasing effects of associated with environmental effects in the wild [47, 48]. We worked with a laboratory setup as we were interested in manipulating specific proximate factors affecting behaviour under controlled conditions rather than studying functional questions, which would warrant field studies [49]. We used both juveniles (3^rd^-4^th^ instar) and adults of the third laboratory generation for the two experiments that we describe below. In experiment 1, we measured (a) nutritional preferences of juveniles and adults, and (b) nutritional intakes of juveniles and adults forced on imbalanced diets (high-carbohydrate vs. high-protein diets, see below). The latter question was addressed by a sub-experiment assessing whether crickets experience nutrient deficiency (or excess) under imbalanced diets. In experiment 2, we subjected freshly emerged nymphs of the 3^rd^ laboratory generation to forced diet treatments to test for the effects of diet on various phenotypic traits. Stock and treatment individuals (eggs, nymphs and adults) were all maintained at 26 °C with 60% relative humidity under a 14 L:10D photoperiod. Nymphs and adults were kept separately in groups of 30 individuals housed in transparent plastic containers (23 × 15 × 17 cm). Containers contained pieces of egg carton for shelter, a plastic water bottle plugged with cotton wool, and a dry bird food (Aleckwa Delikat, Germany) in the case of stock individuals.

We created artificial diets consisting of 40% cellulose and 60% nutrient content by manipulating protein and carbohydrate content following Ref. [50]. Protein consisted of a 3:1:1 mixture of casein, albumen, and peptone, and carbohydrates of a 1:1 mixture of sucrose and dextrin. All artificial diets contained Wesson’s salts (2.5%), ascorbic acid (0.275%), cholesterol (0.55%) and a mix of vitamins (0.18%).

### Experiment 1 – tests of diet preference

Our main study aim was to quantify differences in mean and variance components between two alternative diet treatments (detailed below). The interpretation of associated results warrants information on i) the nutritional preferences for macronutrients (e.g., protein and carbohydrate) in the study species (experiment 1-1), and ii) intake of macronutrients in the imbalanced diet treatments (high-carbohydrate vs. high-protein diets, see below) (experiment 1-2). Crickets can suffer a deficit of protein or an excess of carbohydrate when faced with high-carbohydrate imbalanced diet (see Fig. 1 in [51]) but such information was not available for our study species. We therefore designed an experiment consisting of two sub-experiments, aimed at quantifying 1) nutritional preferences and 2) nutritional intakes (of juveniles and adults) of crickets faced with imbalanced diets.

#### Experiment 1-1. Nutritional preference test

To measure nutritional preferences for carbohydrate and protein by juveniles, 10 juveniles (3^rd^-4^th^ instar) were collected from the stock population 2 weeks after hatching. Each cricket was housed individually in a transparent plastic container (23 × 15 × 17 cm) furbished with pieces of egg carton as shelters, a plastic water bottle plugged with cotton wool, and provided with two dishes. The first dish contained an artificial diet (weighing about 500 mg) containing a 1:29 C:P ratio, while the second dish contained the reverse ratio (29:1) of the same weight. Because juveniles and adults subjected to these nutritional preference tests were taken from our stock populations, and thus previously fed on a dry bird food (see above), we expected them to require some time to adjust to the synthetic diet. We gave them (five to) seven days of acclimation time to the novel diets before collecting preference data. After the acclimation period, we removed both dishes, weighed their dry mass to determine food consumption, and replaced them with fresh food. This procedure was subsequently repeated with a 4-day inter-test interval four times in total. Foods were dried in a desiccating oven at 40 °C before introduction and after removal; consumption rate was defined as the difference between the before and after weight.

Similarly, to measure carbohydrate and protein intake by adults, 37 freshly emerged adult crickets (19 males and 18 females) were allocated to these two complementary foods (two separate food dishes: 29:1 C:P and 1:29 C:P). Each cricket was placed in the same condition as described above for the diet preference test of juveniles. After a week of acclimation, dry mass of food consumed (mass change in the food) was recorded 2 times with a 3-day inter-test interval.

#### Experiment 1-2. Nutritional intake test under imbalanced diets

To assess whether juveniles or adults experienced nutrient deficiency or excess when faced with an imbalanced dietary condition (see Fig. 1 in [51]), we measured the amount of nutrients (carbohydrate and protein) consumed by an individual in a high-carbohydrate (5:1 C:P) or high-protein (1:5 C:P) diet. 12 juveniles, 5 adult males and 5 adult females were allocated to the high-carbohydrate (5:1 C:P) diet, whereas 12 further juveniles, 5 further adult males and 5 further adult females were allocated to the high-protein (5:1 C:P) diet. We used the same procedures to measure the consumption rate as detailed above.

### Experiment 2 – testing phenotypic effects of diet

**Fig. 2:**
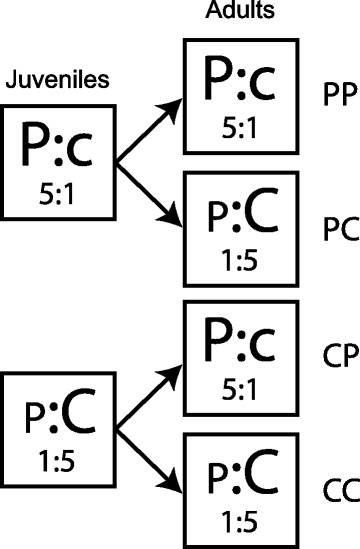
Experimental design used to manipulate diets during juvenile and adult stages: high-carbohydrate juvenile diet & high-carbohydrate adult diet (CC), high-carbohydrate juvenile diet & high-protein adult diet (CP), high-protein juvenile diet & high-carbohydrate adult diet (PC) and high-protein juvenile diet & high-protein adult diet (PP)

This experiment was designed to test how juvenile and adult diet affected phenotypes and their variance components of both sexes. Using approaches detailed in the first section of the methods, we created two different diets: a high-protein (1:5 C:P) versus high-carbohydrate diet (5:1 C:P). We then provided the crickets with an experimental diet using a two-way factorial design implemented as a split-brood design, resulting in 4 groups (CC: high-carbohydrate juvenile and high-carbohydrate adult diet; CP: high-carbohydrate juvenile and high-protein adult diet; PC: high-protein juvenile and high-carbohydrate adult diet; PP: high-protein juvenile and high-protein adult diet; Fig. 2). To do so, eggs were collected from our 3^rd^ generation of the stock population (see above). Once nymphs from the stock population had hatched, they were randomly assigned to a high-carbohydrate or a high-protein juvenile diet, and raised as a group of 30 individuals in transparent plastic containers (23 × 15 × 17 cm) (for animal husbandry conditions, see above). Upon reaching their last instar, individuals were checked daily to determine date of eclosion to adulthood. On the day of eclosion, adults were randomly assigned to the high-carbohydrate or the high-protein adult diet treatment. Each adult individual was maintained in an individual container with an egg carton for shelter and supplied with water and the allocated diet *ad libitum*, which we replaced every 3 days.

When individuals had received 4 weeks of adult diet treatment, we performed a set of behavioural assays, resulting in a total sample size of 109 treated males (CC:17, CP:15, PC:30, PP:47), and 142 treated females (CC:21, CP:27, PC:48, PP:46); we measured exploration, aggression and mating activity. Prior to the initiation of the behavioural assays, each male was identified with a small dot of (Testors enamel) paint on its pronotum. On the same day, exploration, aggression and mating activity were measured in a fixed order with 1-3 minutes between each test. The fixed order of the assays ensured that all individuals experienced the exact same conditions, which facilitates comparison between individuals [52, 53]. Each individual was assayed for each of these 3 behaviours 4 times with a 2-day test-interval. Based on published power analyses [11], this study design (i.e., number of repeats per individual) yielded high enough statistical power to detect low levels of repeatability. All the behavioural assays were recorded with a digital camcorder and analysed with tracking software, Noldus Ethovision XT 10 (Noldus Information Technology).

All assays were performed on a rack fitted with 2 shelves, each equipped with a camera, in the same climate room where the individuals had been reared. Before the assays were conducted, we randomly selected 4 males and 4 females, and randomly assigned 2 males and 2 females per shelf. Each batch of 8 individuals was subsequently assayed simultaneously (2 males and 2 females per shelf) in a fixed order (exploration, aggression and mating activity). Within a batch, all four individuals were first simultaneously subjected to an exploration assay alone (Additional file 1: Figure S2). Ten minutes following the onset of the exploration assay, the 2 same-sex individuals of the same shelf were placed together in a single arena to measure aggressiveness (Additional file 1: Figure S2). Ten minutes following the onset of the aggression assay, the 2 same-sex individuals were separated, and the male and female of the same shelf placed together in the same arena to assay mating activity (Additional file 1: Figure S2). For the aggression and mating behaviour tests, we randomly assigned individuals into dyads (see above) without considering their diet treatments. As each individual was repeatedly assayed but not always with the same partner, our study design was perfectly suited to estimate how much of the phenotypic variance expressed by focal individuals (in aggression or mating behaviour) was attributable to the identity of the focal individual, the identity of its social partner, or residual variance [34, 54–56]; partner identity effects estimated by our study design represented the combined influences of indirect genetic and indirect permanent environmental effects [57–59]. Fifteen minutes following the onset of the mating activity assay, all individuals of the batch were returned to their private rearing containers. Following the execution of all assays within a batch, the testing arenas were thoroughly cleaned and sand was exchanged for fresh sand to minimize any possible effects of remaining contact pheromones.

#### Novel environment assays

Individuals were removed from their individual containers and placed in a plastic arena with a removable partition in the middle which created two small rooms (15 × 15 × 10 cm, Additional file 1: Figure S2). The two individuals were separated by an opaque partition in the middle of the arena during the novel environment assay (Additional file 1: Figure S2). In each compartment, fine-grained white sand was spread on the bottom, and a plastic semi-cylinder was provided as a shelter (Fig. 2 in [55]). The tracking software then measured each individual’s total distance moved in the compartment for 10 minutes [55, 60]. The compartment was novel to the crickets, and we essentially quantified how much time individuals spend out of the shelter, exploring the novel environment. We therefore labelled this behavioural variable ‘exploration behaviour’ [61].

#### Aggression assays

Aggression assays began when the shelters and the partition in the middle of the arena were removed 10 minutes after the onset of the exploration assay (Additional file 1: Figure S2). Two same-sex individuals then interacted and showed behaviour ranging from low-level aggression (e.g., antennal fencing, threat postures) to high-level aggression (e.g. aggressive song stridulation, flaring mandibles and biting) [62]. Each aggression test lasted for 10 minutes. The focal and opponent identities were randomly assigned after the experiment for statistical analyses. The tracking software measured the duration of attack when the focal individual chased the opponent (within 6 cm) to attack it; this measure represents an appropriate proxy for aggressiveness based on our previous work on this species [60].

#### Mating activity assays

Following the aggression assays (performed for males and females simultaneously tough separately), we gently moved both a male and a female into the same plastic arena (15 × 15 × 10 cm) and left both to acclimatize for 30 seconds. The arena was of the same size as the one used for the novel environment assay. During such tests, once males recognized females, they typically start courting females using courtship stridulation [63, 64]. Once a female was attracted and mounted the courting male, the male would normally attempt to transfer his spermatophore. Following copulation, the male typically remains close to the female to increase the duration of spermatophore attachment [65] and attempts to remate [66, 67]. Therefore, how closely the male remained in the proximity of the female indicates how actively the male courts before, and guards after, copulation. We thus measured 1) the average distance between the body centre of the male and the body centre of the female as a proxy for male mating activity, which we did for 15 minutes, and 2) female latency to mount as a proxy for female mating activity. Females who did not respond to males producing courtship stridulation were given a mating activity (i.e., female latency) score of 15 mins (26% of the total number of female responses were scored as 15 mins). Notably, females do not approach males that fail to produce courtship stridulation; we therefore did not analyse female mating activity data when the male did not produce courtship song as this typically resulted in a failure of a female to mount a male.

#### Morphological measures

We weighed each individual to the nearest 0.001 g at the end of the second and the fourth set of behavioural assays. Individuals were euthanized by placing them in a -20 °C freezer for 24 h following the last set of behavioural tests, dried at approximately 50 °C for 48 h, and weighed. We then extracted lipids from the dried crickets using two 24 h washes of chloroform [68], and samples were again dried for 24 h and weighed. Lipid masses were calculated by subtracting sample lean (lipid extracted) dry masses from sample dry masses.

### Statistical methods

#### Experiment 1 – Tests of diet preference

We used unpaired t-tests to test for differences in 1) carbohydrate intake, 2) protein intake and 3) total nutrient intake (total consumption of carbohydrate and protein) between treatments or sexes.

#### Experiment 2- Diet effects on mean phenotype

To calculate the effect of diet on an individual’s mean phenotype (assessed for exploration, aggression, mating activity and body weight), we used univariate mixed-effects models including juvenile diet treatment (high-carbohydrate diet as the contrast), adult diet treatment (high-carbohydrate diet as the contrast), their interaction, sex (females as the contrast), interactions between diet treatments and sex, time of the day (hour), testing order, and shelf (a 2-level factor, upper and lower shelves) as fixed effects. Time of the day was mean-centred at the population level. We also fitted individual identity as a random effect; partner identity was also included as an additional random effect in the analyses of aggression and mating activity (following [34, 54]). Prior to analysis, response values were standardised (mean = 0, SD = 1) to ease interpretation. We assessed the significance of fixed effects using Wald F-tests, and the significance of random effects using likelihood ratio tests (LRTs). The test statistic associated with the LRT was calculated as twice the difference in log likelihood between models with vs. without a random effect. To test the effect of the focal individual’s identity or the partner individual’s identity, the value of P was calculated using a mixture of P(χ^2^, df = 0) and P(χ^2^, df = 1) [69–71].

Because we measured lipid mass of individuals once, we did not acquire repeated measures for this trait. For its analysis, we therefore used a general linear model, where we included juvenile diet treatment, adult diet treatment, their interaction, sex, interactions between diet treatments and sex, and testing order as fixed effects. We assessed the significance of fixed effects using Wald F-tests.

Additionally, since we randomly paired individuals in the aggression and mating behaviour assays without considering their diet treatments, we additionally included the fixed effects of (i) the partner’s treatment and (ii) the interaction between the focal and partner treatment on the focal individual’s behaviour for analyses of aggressiveness and mating behaviour.

#### Experiment 2- Diet effects on variance components and repeatabilities

To test for diet effects on among- and within-individual variances, total phenotypic variances or repeatabilities, we used multivariate mixed-effects models which simultaneously fitted the same trait measured in the four within-sex treatment groups (CC, CP, PC or PP (Fig. 2); subscripts M vs. F used for males vs. females) as the four response variables (e.g., EXP_CC(M)_, EXP_CP(M)_, EXP_PC(M)_, EXP_PP(M)_ for the male dataset: exploration of males reared under the CC/CP/PC/PP treatment). We fitted covariates (time of the day or testing repeat) as fixed effects. Depending on the response variable, we also included the focal individual’s identity, or both the focal and partner individual identities (for aggression and mating activity), as random effects.

In models built to test for diet effects on variance components (i.e., among-individual variances (V_I_), within-individual variances (V_R_) or total phenotypic variances (V_P_)), we used the untransformed trait values as response variables. In models fitted to test diet effects on repeatabilities, we used z-transformed trait values (i.e., mean = 0 and SD = 1) instead. As repeatability is calculated as the V_I_ divided by V_P_, V_I_ represents the repeatability for z-transformed data (i.e., where V_P_ = 1) [11]. In the multivariate mixed-effect model, due to the study’s design, covariance parameters were not estimable and therefore constrained to zero at all levels.

To test for the significance of diet effects on V_I_, V_R_, V_P_ or repeatability, we compared the multivariate model, which fitted treatment-specific variances at the focal identity level (detailed above; model 2-4, Table 1), with a reduced (i.e., ‘null’) model in which the focal variance component was constrained to be equal across the four datasets (model 1 where V_CC_ = V_CP_ = V_PC_ = V_PP_). To explore whether juvenile diet affected variance components or repeatabilities, we compared our null model (model 1) with a model (model 2) where variances were constrained to be equal for treatments sharing the same juvenile diet (i.e., [V_CC_ = V_CP_] ≠ [V_PC_ = V_PP_]). Similarly, to test whether adult diets affected variance components or repeatabilities, we compared a null model (model 1) with a model (model 3) where variances were constrained to be equal for treatments sharing the same adult diet (i.e., [V_CC_ = V_PC_] ≠ [V_CP_ = V_PP_]). Finally, to test whether juvenile and adult diet treatments were both important (either due to additive or interactive effects), we compared a null model (model 1) with a heterogeneous model where all variances were free to vary (model 4). To test whether diet treatment affected the total phenotypic variance, we removed all random effects from the model and then compared the residual (i.e., phenotypic) variance among treatments as detailed above. We compared models using likelihood ratio tests (LRTs). As an alternative approach, we also compared models based on their Akaike Information Criterion (AIC) weights (a measure of relative support for each *a priori* considered model structure) [72, 73]. AIC weight is the probability that the candidate model would be the best fitting model among the set of models considered. Because both approaches led to the same conclusions, we used results from LRTs in the main text (Table 3), and provide the analysis based on AIC weights as supplementary material (Table S1). All the statistical analyses were implemented in ASReml 3.0 and solved by restricted maximum likelihood.

**Table 1:** Overview of the series of multivariate mixed-effect models fitted and compared to estimate diet effects on individual differentiation in behaviour and within-individual behavioural stability

Model	Variance structures	Model description
Model 1 (M1)	V_CC_ = V_CP_ = V_PC_ = V_PP_	Null model – Homogeneity of variance components across all diet treatments
Model 2 (M2)	[V_CC_ = V_CP_] ≠ [V_PC_ = V_PP_]	Effect of juvenile diet - V was constrained the same within the same juvenile diet treatments
Model 3 (M3)	[V_CC_ = V_PC_] ≠ [V_CP_ = V_PP_]	Effect of adult diet - V was constrained the same within the same adult diet treatments.
Model 4 (M4)	V_CC_ ≠ V_CP_ ≠ V_PC_ ≠ V_PP_	Additive/non-additive effect of juvenile and adult diet -Unconstrained model.

Models differ in whether treatment-specific variance components (V) were estimated as distinct or constrained to be identical. This procedure was applied to study treatment effects on either among-individual (V_I_) or within-individual variances (V_R_)

## Results

### Experiment 1 - Tests of diet preference

#### Experiment 1-1

**Fig. 3:**
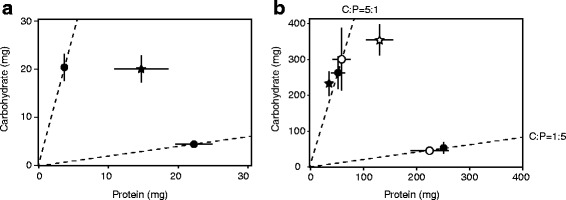
Carbohydrate and protein intake of **a** juveniles, and **b** adult males and females. In both panels, stars indicate intake targets when adult individuals are given a choice between nutritionally complementary diets (juvenile: closed star in (**a**); adult male: closed star in (**b**); adult female: open star in (**b**)). In panels, circles indicate the nutrient intake when individuals (juvenile: closed circle in (**a**); adult male: closed circle in (**b**); adult female: open circle in (**b**)) are given an imbalanced diet (either 5:1 C:P or 1:5 C:P). Error bars represent standard errors but too small errors are not visible in the figure. Dotted lines represent the experimental high-carbohydrate (5:1) and high-protein (1:5) diets

The ratio of average carbohydrate consumption to average protein consumption (C:P) by juveniles over an entire week (e.g. [60]) was 1:1.3. This finding implied that 2-week-old juveniles preferred to eat relatively similar amounts of carbohydrates (mean ± SE: 20 ± 3 mg) versus proteins (15 ± 4 mg; Fig. 3a). Adults of both sexes, by contrast, consumed relatively more carbohydrates: male diet preference (i.e., C:P ratio) was 5.7:1, and female diet preference was 2.7:1. When adults were able to choose and consume both a high-carbohydrate and a high-protein diet simultaneously, adult males consumed less carbohydrates (*t*_35_ = -3.14, *P* = 0.003, Fig. 3b) and less protein (*t*_35_ = -3.60, *P* < 0.001, Fig. 3b) than females. Altogether, males therefore consumed less nutrients (calculated as the summed total of carbohydrate and protein consumption) compared to females (*t*_35_ = -3.54, *P* = 0.001).

#### Experiment 1-2

Crickets of both age classes consumed less carbohydrates and more protein compared to their preferred intake when forced on an imbalanced high-protein (i.e., 1:5 C:P) diet (Fig. 3a,b). Juveniles and adult females consumed less protein but similar amounts of carbohydrates compared to their preferred intake when forced on a high-carbohydrate diet (5:1 C:P) (Fig. 3a,b). By contrast, adult males forced on a high-carbohydrate diet (5:1 C:P), had intake rates of both macronutrients that were close to their intake target (i.e., 5.7:1 C:P, see above; Fig. 3b) as adult males generally prefer relatively carbohydrate-biased diets.

### Experiment 2 – diet effects on mean phenotype

Juvenile diet strongly affected morphological and behavioural traits of both sexes other than exploration behaviour and female mating activity (Table 2; Additional file 1: Figure S3). Individuals raised on high-protein juvenile diets were more aggressive and heavier as adults compared to individuals raised on high-carbohydrate juvenile diets (Table 2; Additional file 1: Figure S3). Males raised on high-protein juvenile diets also courted and guarded females more closely compared to males raised on high-carbohydrate juvenile diets (Table 2; Additional file 1: Figure S3). Given the way that we set up the experiment (see Methods), this latter finding implied either (i) that juvenile diet affected both aggressiveness and mating behaviour, or (ii) that mating behaviour was affected by carry-over effects associated with aggressive individuals winning fights (see Discussion).

**Table 2:** Linear (mixed) models of behavioural and morphological traits as a function of diet, sex and their interaction. Parameters are provided with standard errors in parentheses

	Exploration	Aggression	Male mating activity	Female mating activity	Weight	Lipid mass
*Fixed effects*						
Juvenile diet (J)^a^	β = 0.10(0.21) F_1,244.7_ = 0.21 *P* = 0.64	β = **0.51(0.21)** F_1,224_ = 13.48 *P* < 0.001	β = **0.39(0.17)** F_1,102.1_ = 13.78 *P* < 0.001	β = 0.03(0.19) F_1,126.4_ = 0.70 *P* = 0.40	β = **0.82(0.18)** F_1,239.4_ = 54.81 *P* < 0.001	β = **1.14(0.24)** F_1,233_ = 22.00 *P* < 0.001
Adult diet (A)^b^	β = 0.01(0.23) F_1,242.9_ = 0.22 *P* = 0.63	β = 0.17(0.24) F_1,216.2_ = 2.06 *P* = 0.16	β = -0.25(0.21) F_1,111.2_ = 1.49 *P* = 0.23	β = 0.17(0.20) F_1,119.4_ = 0.01 *P* = 0.93	β = **0.80(0.21)** F_1,239.1_ = 30.39 *P* < 0.001	β = **-0.14(0.26)** F_1,233_ = 31.34 *P* < 0.001
Sex^c^	β = **1.03(0.26)** F_1,242.4_ = 48.07 *P* < 0.001	β = 0.43(0.28) F_1,209.2_ = 10.72 *P* < 0.001	NA	NA	β = **-0.78(0.23)** F_1,239_ = 169.97 *P* < 0.001	β = **-0.12(0.15)** F_1,233_ = 33.39 *P* < 0.001
J × A^d^	β = -0.01(0.28) F_1,244.3_ = 1.39 *P* = 0.24	β = -0.41(0.29) F_1,291.4_ = 0.84 *P* = 0.36	β = 0.14(0.25) F_1,104.3_ = 0.29 *P* = 0.59	β = -0.24(0.24) F_1,125.7_ = 1.01 P = 0.32	β = -0.17(0.25) F_1,239.6_ = 0.76 *P* = 0.38	β = -0.62(0.32) F_1,233_ = 2.81 *P* = 0.08
J × Sex	β = -0.43(0.31) F_1,244.1_ = 0.19 *P* = 0.66	β = 0.002(0.33) F_1,220.2_ = 1.40 *P* = 0.24	NA	NA	β = -0.05(0.28) F_1,239.4_ = 0.05 *P* = 0.82	β = **-0.75(0.36)** F_1,233_ = 4.13 *P* = 0.04
A × Sex	β = -0.53(0.36) F_1,242.7_ = 0.17 *P* = 0.67	β = -0.45(0.37) F_1,214.9_ = 0.28 *P* = 0.60	NA	NA	β = **-0.45(0.33)** F_1,239_ = 5.94 *P* = 0.02	β = -0.31(0.41) F_1,233_ = 0.00 *P* = 0.95
J × A × Sex	β = 0.63(0.43) F_1,243.9_ = 2.09 *P* = 0.52	β = 0.50(0.45) F_1,213.6_ = 1.24 *P* = 0.27	NA	NA	β = 0.007(0.39) F_1,239.4_ = 0.00 *P* = 0.99	β = 0.47(0.50) F_1,233_ = 0.91 *P* = 0.34
Time of day	β = **-0.03(0.01)** F_1,776.6_ = 6.69 *P* = 0.01	β = **-0.05(0.02)** F_1,325.5_ = 4.85 *P* = 0.03	β = **-0.05(0.02)** F_1,282.3_ = 4.55 *P* = 0.04	β = **-0.10(0.03)** F_1,298.5_ = 14.9 *P* < 0.001	NA	NA
Testing order	β = **-0.02(0.009)** F_1,729.9_ = 6.81 *P* = 0.01	β = -0.03(0.02) F_1,293.7_ = 3.23 *P* = 0.08	β = **-0.04(0.02)** F_1,232.2_ = 4.50 *P* = 0.04	β = 0.006(0.02) F_1,238.1_ = 0.08 *P* = 0.77	β = **0.02(0.005)** F_1,239.8_ = 10.23 *P* = 0.002	NA
Shelf	β = -0.01(0.04) F_1,796.5_ = 0.02 *P* = 0.89	β = -0.04(0.08) F_1,396.2_ = 0.26 *P* = 0.60	NA	NA	NA	NA
Intercept	β = -0.41(0.17) F_1,267.5_ = 0.72 *P* = 0.40	β = -0.50(0.18) F_1,255.4_ = 0.27 *P* = 0.60	β = -0.26(0.15) F_1,128_ = 0.66 *P* = 0.42	β = -0.13(0.17) F_1,117.5_ = 2.48 *P* = 0.12	β = -0.45(0.15) F_1,244.8_ = 0.49 *P* = 0.48	β = -0.20(0.20) F_1,233_ = 0.00 *P* = 1.00
*Random effects*						
ID	0.51 (0.06)	0.25 (0.06)	0.14 (0.05)	0.04 (0.05)	0.88 (0.03)	NA
Partner ID	NA	0.20 (0.06)	0.18 (0.06)	0.19 (0.06)	NA	NA
Residual	0.36 (0.02)	0.51 (0.06)	0.66 (0.07)	0.76 (0.08)	0.10 (0.01)	0.71 (0.03)

^a^juvenile diet effect (high-carbohydrate diet as the contrast)

^b^adult diet effect (high-carbohydrate diet as the contrast)

^c^sex effect (females as the contrast)

^d^interactive effect between juvenile and adult diets

Significant terms are indicated in bold

While juvenile diet treatment affected various behaviours, this was not the case for adult diet treatment: adult diet affected only body weight and lipid mass (Table 2; Additional file 1: Figure S3). Individuals forced on a high-protein adult diet gained more weight but had lower lipid mass compared to those forced on a high-carbohydrate adult diet (Table 2; Additional file 1: Figure S3). Across diet treatments, males were more aggressive and explorative in a novel environment than females (Table 2). Females were heavier and their bodies contained more lipids than males (Table 2). There was no evidence for significant sex differences in the effects of juvenile or adult diet treatment for any phenotypic trait except for body weight and lipid mass (Table 2; Additional file 1: Figure S3): Both sexes had more lipids in their body when they were reared on the high-protein juvenile diet but the difference in body lipids between juvenile diet treatments was larger in females compared to males. Similarly, both sexes gained more weight when they were forced on the high-protein adult diet but the difference in body weight between adult diet treatments was larger in females compared to males.

For the interactive phenotypes (aggression and mating activity), the partner’s diet treatment affected aggression and mating behaviour in females (Additional file 1: Table S3, female aggression, F_4,138.7_ = 3.10, *P* = 0.02; female mating activity, F_4,123.3_ = 3.19, *P* = 0.02). Females tended to be more aggressive and more active in the mating assay when they encountered a partner that had received a high-protein compared to a high-carbohydrate adult diet. However, the effect of partner treatment did not affect the significance of effects of other factors (Additional file 1: Table S3).

### Experiment 2 – diet effects on variance components and repeatabilities

**Fig. 4:**
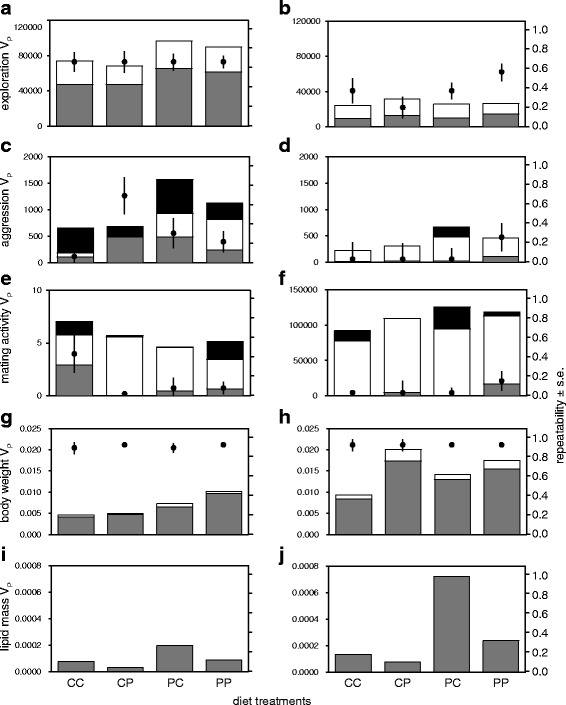
Variance components of phenotypes across four combinations of diet treatments (CC, high-carbohydrates juvenile & high-carbohydrates adult diet treatment; CP, high-carbohydrates juvenile & high-protein adult diet treatment; PC, high-protein juvenile & high-carbohydrates adult diet treatment; PP, high-protein juvenile & high-protein adult diet treatment). **a** male exploration, **b** female exploration, **c** male aggression, **d** female aggression, **e** male mating activity, **f** female mating activity, **g** male body weight, **h** female body weight, (**i**) male lipid mass and **j** female lipid mass. Stacked bars (y-axis, *left*) indicate the total phenotypic variance decomposed into its among-individual (*dark grey bars*), residual within-individual (*white bars*) variance components and a variance explained by the interacting partner individual (*black bars*). Dots (y-axis, right) represent level of repeatability (± s.e.)

We estimated the among-individual, within-individual, and total phenotypic variance for each unique combination of juvenile and adult treatment and phenotypic trait (for age-, treatment-and trait-specific estimates of each variance component see Table S2 in the supplementary material). Diet affected the expression of the total phenotypic, among- and/or within-individual variances of various traits (Fig. 4; Table 3). However, diet treatment did not simultaneously affect among- and within-individual variances such that it significantly changed any of the trait repeatabilities (Table 4). Diet treatment affected the total phenotypic variance for aggression, body weight and lipid mass in both sexes (Fig. 4; Table 3). For aggression and lipid mass, the high-carbohydrate adult diet increased the total phenotypic variance when crickets were reared on a high-protein juvenile diet (Fig. 4). This effect was not observed when crickets were reared on a high-carbohydrate juvenile diet (Fig. 4). In contrast, differences in the total phenotypic variance in female body weight between adult diet treatments were larger when juveniles were raised on high-carbohydrate than on high-protein diets (Fig. 4).

**Table 3:** Diet effects on variance components (V_I_, among-individual variance; V_R_, within-individual variance; V_P_, total phenotypic variance) and repeatability (*R*) of exploration, aggression, mating activity, weight and lipid mass)

						Males					
		Exploration		Aggression		Mating activity		Weight		Lipid mass	
	Diet effect	χ^2^_df_	*P*	χ^2^_df_	*P*	χ^2^_df_	*P*	χ^2^_df_	*P*	χ^2^_df_	*P*
V_I_	Juvenile	0.66_1_	0.42	0.06_1_	0.80	1.69_1_	0.19	**3.74**_1_	**0.05**	NA	NA
	Adult	0.00_1_	1.00	0.23_1_	0.63	2.17_1_	0.14	1.81_1_	0.18	NA	NA
	Combined	0.68_3_	0.88	1.05_3_	0.79	**9.03**_3_	**0.03**	5.03_3_	0.17	NA	NA
V_R_	Juvenile	1.26_1_	0.26	**4.68**_1_	**0.03**	0.12_1_	0.73	1.34_1_	0.25	NA	NA
	Adult	0.50_1_	0.48	0.04_1_	0.85	0.88_1_	0.35	2.87_1_	0.09	NA	NA
	Combined	2.22_3_	0.53	6.86_3_	0.08	2.48_3_	0.48	5.64_3_	0.13	NA	NA
V_P_	Juvenile	3.60_1_	0.06	**6.72**_**1**_	**0.01**	3.44_1_	0.06	**13.2**_1_	**<0.001**	**8.64**_1_	**<0.001**
	Adult	0.08_1_	0.78	2.36_1_	0.12	0.20_1_	0.65	2.96_1_	0.09	**8.96**_1_	**<0.001**
	Combined	3.84_3_	0.28	**10.74**_**3**_	**0.01**	4.08_3_	0.25	**20.24**_3_	**<0.001**	**17.32**_3_	**<0.001**
*R*	Juvenile	0.002_1_	0.96	1.32_1_	0.25	0.77_1_	0.38	0.00_1_	1.00	NA	NA
	Adult	0.01_1_	0.92	0.03_1_	0.87	1.99_1_	0.16	0.02_1_	0.89	NA	NA
	Combined	0.05_3_	1.00	1.49_3_	0.69	4.45_3_	0.22	0.02_3_	1.00	NA	NA
						Females					
		Exploration		Aggression		Mating activity		Weight		Lipid mass	
	Diet effect	χ^2^_df_	*P*	χ^2^_df_	*P*	χ^2^_df_	*P*	χ^2^_df_	*P*	χ^2^_df_	*P*
V_I_	Juvenile	0.04_1_	0.84	1.18_1_	0.28	0.76_1_	0.38	0.08_1_	0.78	NA	NA
	Adult	1.16_1_	0.28	1.05_1_	0.31	1.94_1_	0.16	1.63_1_	0.20	NA	NA
	Combined	1.24_3_	0.74	1.78_3_	0.62	2.64_3_	0.45	2.84_3_	0.42	NA	NA
V_R_	Juvenile	1.76_1_	0.18	2.70_1_	0.10	0.54_1_	0.46	0.47_1_	0.49	NA	NA
	Adult	0.34_1_	0.56	0.44_1_	0.51	0.40_1_	0.53	**6.94**_1_	**0.01**	NA	NA
	Combined	5.66_3_	0.13	3.51_3_	0.32	0.00_3_	1.00	**8.20**_3_	**0.04**	NA	NA
V_P_	Juvenile	0.24_1_	0.62	**14.58**_1_	**0.001**	1.08_1_	0.30	0.06_1_	0.81	**50.64**_1_	**0.001**
	Adult	0.84_1_	0.36	2.96_1_	0.09	0.14_1_	0.71	**5.18**_1_	**0.02**	**36.84**_1_	**0.001**
	Combined	1.58_3_	0.66	**20.24**_3_	**0.001**	1.56_3_	0.67	**9.10**_3_	**0.03**	**71.8**_3_	**0.001**
*R*	Juvenile	0.11_1_	0.74	0.40_1_	0.53	0.53_1_	0.47	0.00_1_	1.00	NA	NA
	Adult	0.98_1_	0.32	0.92_1_	0.34	2.51_1_	0.11	0.06_1_	0.81	NA	NA
	Combined	1.13_3_	0.77	1.24_3_	0.74	2.96_3_	0.40	0.10_3_	0.99	NA	NA

χ^2^-values were derived from likelihood ratio tests (see Methods)

Significant effects are indicated in bold

**Table 4:** Within-treatment repeatabilities of behaviours and body weight with standard errors in parentheses

R (SE)	Exploration	Aggression	Mating activity	Body weight
Male *R*_adj_	**0.68 (0.04)**	**0.30 (0.09)**	**0.14 (0.05)**	**0.95 (0.01)**
CC	**0.66 (0.11)**	0.16 (0.23)	**0.40 (0.18)**	**0.89 (0.05)**
CP	**0.68 (0.12)**	**0.64 (0.18)**	0.00 (0.00)	**0.95 (0.03)**
PC	**0.67 (0.08)**	**0.30 (0.15)**	0.11 (0.09)	**0.90 (0.04)**
PP	**0.68 (0.06)**	0.21 (0.13)	0.13 (0.07)	**0.95 (0.01)**
Female *R*_adj_	**0.43 (0.05)**	0.12 (0.08)	0.04 (0.05)	**0.91 (0.01)**
CC	**0.39 (0.14)**	0.05 (0.22)	0.00 (0.00)	**0.90 (0.05)**
CP	0.20 (0.12)	0.08 (0.20)	0.04 (0.15)	**0.87 (0.05)**
PC	**0.38 (0.08)**	0.03 (0.14)	0.02 (0.08)	**0.92 (0.02)**
PP	**0.54 (0.08)**	0.23 (0.14)	0.14 (0.08)	**0.89 (0.03)**

An average adjusted repeatability (*R*_adj_) for the whole dataset is presented, and calculated after including diet treatment as a fixed effect factor into the model. CC, high-carbs juvenile & high-carbs adult diet treatment; CP, high-carbs juvenile & high-protein adult diet treatment; PC, high-protein juvenile & high-carbs adult diet treatment; PP, high-protein juvenile & high-protein adult diet treatment

Variance components at the boundary are estimated as 0.00 with 0.00 SE

Significant values (P < 0.05) are indicated in bold

Males raised on the high-protein juvenile diet exhibited larger within-individual variance in aggression and larger among-individual variance in weight compared to males raised on the high-carbohydrate juvenile diet (Fig. 4, Table 2). Females raised on the high-protein juvenile diet exhibited larger within-individual variance in body weight compared to females raised on the high-carbohydrate juvenile diet (Fig. 4, Table 3). In addition, the among-individual variance in male mating activity differed among four diet combination treatments (Fig. 4, Table 2): when males were reared on the high-carbohydrate juvenile diet, the adult diet containing more carbohydrate increased the among-individual variance in male mating activity while adult diet did not affect the among-individual variance in male mating activity when males were reared on the high-protein juvenile diet (Fig. 4, Table 2).

There were no sex differences in the effect of diet treatment on the total phenotypic variance (Table 3). However, males and females differed in how diet affected among- or within-individual variance components (Table 3). While variance components in female aggression and mating activity were not influenced by diet treatment, the among-individual variance in male mating activity and the within-individual variance in male aggression were diet-dependent (Table 3).

## Discussion

One of the key meta-analytical findings in animal personality research is that about 50% of the individual differences in average behaviour are attributable additive genetic effects [4]. Our study shows that nutritional history partly explains the remaining variation, causing environmentally-underpinned repeatable differences in behaviour. The nutritional environment affected both the amount of individual differentiation and the amount of within-individual stability in various phenotypic traits: four of five traits assayed in males (including two of three behaviours), and three of five traits assayed in females (including one of three behaviours), showed diet-dependent variance components (Table 3). Nutritional history during ontogeny in particular represented an important environmental factor mediating such non-genetic differences in ‘personality’: crickets raised on a high-protein diet developed a more aggressive phenotype in adulthood compared to those raised on a high-carbohydrate diet; males raised on a high-protein diet also courted females more actively in adulthood. Juvenile diet, furthermore, also affected an individual’s level of behavioural stability: males raised on a high-protein diet were not only more aggressive on average but also behaviourally less stable (i.e., more variable or ‘changeable’) compared to males raised on a carbohydrate-rich diet. Furthermore, we found some evidence for interacting effects of early-life and adult diet on the individual differentiation in behaviour, indicating the existence of multidimensional plasticity [74, 75], though such interactive effects were only detected for the individual differentiation in male mating activity. Finally, for some traits, patterns of diet-specific behavioural stability differed between the sexes. The effect of diet on behavioural stability and individual differentiation was relatively weak in females compared to males.

### Diet influences personality and morphology

Our study showed that the nutritional environment at the juvenile stage affected the development of behavioural (namely; aggression and mating activity) and morphological traits (Table 2). Juveniles raised on low-protein and high-carbohydrate (5:1 C:P) diets were not able to reach their preferred intake of proteins (Fig. 3a). Proteins are a limiting nutrient under natural field conditions [76, 77] and strongly affect development [78]. In insects, it is typical that protein deficiency during development causes smaller body size and longer development time [79–81]; malnutrition during development is also known to reduce juvenile and adult body lipid content [43, 82]. In agreement with previous findings, individuals reared on high-protein juvenile diets gained more weight and body lipids, and individuals reared on high-carbohydrate juvenile diets suffered increased mortality (CS Han & NJ Dingemanse, unpublished data). This may explain why males on high-protein juvenile diets were also more aggressive towards conspecifics and more active in courtship and post-copulation mate-guarding (Table 2). Therefore, the behavioural effects of juvenile diet were likely mediated by the acquisition of proteins required for development.

Adult diet also played a role in the expression of morphology but not in the development of a cricket’s personality. Adult diet was not able to offset the detrimental effect of the juvenile high-carbohydrate diet (‘silver-spoon hypothesis’, [83]). When considering adult intake targets (Fig. 3b), males and females clearly suffered carbohydrate deficiency when raised on a high-protein adult diet. In contrast, among animals raised on the high-carbohydrate adult diet, only females suffered protein-deficiency while males instead consumed enough proteins to meet their preferred needs. Because our adult diet treatments were not extreme, individuals confronted with a high-protein adult diet could probably acquire energy also by consuming more protein, which may explain why adult diet generally did not elicit effects on the expression of assayed phenotypic traits.

In addition, given the fixed sequential nature of our experiments (where mating activity was always measured after the aggression assay), we suggest caution in interpreting these results: though diet affected both male aggression and male mating activity, the outcome of the contest might have directly spilled-over to affect mating behaviour. Such carry-over effects of social interactions are generally expected [84], in this particular case possibly mediated by winner and/or loser effects (e.g., [85–87]). If so, we would expect a positive correlation between aggression and mating activity, for example, because less aggressive crickets more readily lose fights, which in turn would inhibit subsequent mating activity. In our experiment, male aggression indeed tended to be positively correlated with male mating activity at the among-individual level (bivariate mixed-effects model: r = -0.40 ± 0.21, *P* = 0.08). This finding is consistent with the notion that aggressive males had higher mating activity because they won fights. However, this positive correlation may also be due to a genetic correlation between aggressiveness and mating behaviour that is not mediated by winner-loser effects. Further experiments, where testing order and inter-test intervals are explicitly manipulated [53, 88, 89], are therefore required to differentiate between these two alternative explanations.

### Diet influences individual differentiation and behavioural stability

In this paper, we provide experimental evidence that nutritional history affects the amount of individuality (i.e., among-individual variance) and behavioural stability (i.e., within-individual variance) of certain behavioural traits. Detected changes in individual differentiation or within-individual stability of phenotypes were related to the juvenile rather than adult diet treatment (Table 2). First, we showed that the high-protein juvenile diet increased the mean level of aggression while simultaneously decreasing its within-individual stability in adulthood (though this latter effect existed only in males). This finding thereby partly supports the hypothesis that both the mean level and variance components (among- and within-individual variances) of phenotypic traits are greater in high-quality (vs. low-quality) environments (Additional file 1: Figure S1).

The following mechanisms may explain the simultaneous increase in mean and level of instability in male aggression. When two males interact, a behavioural hierarchy between them is developed during the fight [90–92]. Following the establishment of a hierarchy, the loser male rarely approaches and attacks the winner male, whereas the winner males actively chases and attacks the loser [90–92]. Males developed in high-quality nutritional environments (i.e., our high-protein juvenile diet, Fig. 3b), where individuals meet their protein needs, may gain more weight and thus be more aggressive. This means that males in a good condition can decide to express low levels of aggressiveness when they lose fights, but they can act hyper-aggressively when they win the fight. This may explain why high-quality nutritional environments can decrease the within-individual stability but increase the mean level of aggression.

Our study also showed that the nutritional environment changes the amount of among-individual differentiation and level of behavioural stability without affecting mean levels of behaviour. For example, despite a combined effect of adult and juvenile diets on the amount of individual differentiation in male mating activity, the mean level of male mating activity was only affected by juvenile diet (Fig. 4). If some individuals increase their behavioural level, but others decrease it, this may result in an increased individual differentiation without changing mean values (Additional file 1: Figure S1d). Since the expression of behaviour is also mediated by trade-offs between life-history traits [93], males facing nutritionally balanced dietary environments (e.g. high-carbohydrate adult diets) may vary their reproductive investment using abundant resources. That is, some males may invest more in current reproduction and increase the intensity of mating activity while others instead decrease their current investment in reproduction and mating activity. We thus suggest that the extent of individual differentiation and behavioural stability can change regardless of changes in mean value. Taken together, our study shows that individual differentiation in behaviour and behavioural stability were independently responding to diet regardless of the effect on the population-level mean (Additional file 1: Figure S1). The effects of diet on personality and behavioural stability were thus trait-specific (Table 3).

This study investigated the underlying proximate mechanisms causing variation in among-individual differentiation and within-individual behavioural stability among datasets. Our findings imply that environmental conditions such as diet can indeed explain the existence of variation in these variance components, clarifying why those may vary across datasets. High-quality environments such as nutritionally balanced and pathogen-free conditions may enable individuals in a good condition to flexibly express costly behaviours in response to changes in environments. An increase in resource availability (e.g. protein) characterizing high quality environments may also increase among-individual differences in how trade-offs (e.g., between lifespan and reproduction) are resolved, thereby leading to further among-individual differentiation in behaviour [21].

Our results are in line with previous research showing that the expression of genetic variation is typically increased under favourable conditions [19, 20], and recent work by Royaute and Dochtermann [16] showing that nutritional stress from low quality diet increased the within-individual stability in the response to predators in the house crickets (*Acheta domesticus*). By contrast, some recent studies showed results opposite to ours: the amount of individual differentiation in behaviour was higher, and within-individual behavioural stability lower in more stressful environments [29]. Hermit crabs (*Pagurus bernhardus*) also reduced their within-individual behavioural stability in the presence of predators (i.e., a stressful environment) because unpredictability in behaviour under increased perceived predation risk might decrease their actual predation risk [13]. Recent work on zebra finches (*Taeniopygia guttata*) also showed that early dietary restriction increased the amount of among-individual variance in activity [14]. These lines of contrasting evidence imply that the environment may directly affect the amount of individual differentiation in behaviour and within-individual behavioural stability but that such effects might often be trait-specific. Taken together, while our study documents diet effects on variance components, the contrasting evidence and trait-specific effects suggest that further studies of this kind are required to assess the generality of our findings across species.

### Diet effects on repeatability

As repeatability is calculated as the proportion of the among-individual variance relative to the total phenotypic variance [94], a significant change in the amount of among-individual variation, or level of behavioural stability, is expected to cause a change in repeatability. However, despite diet affecting the extent of individual differentiation and level of behavioural stability, we failed to find significant effects of diet on behavioural repeatability (Table 3). Here we suggest explanations for this finding. Firstly, diet may have little influence on the repeatability when it affects a variance component that accounts for a relatively small part of the total phenotypic variance. For example, when the amount of within-individual variance is much larger than the amount of among-individual variance, only a large change in among-individual variance might lead to a statistically detectable change in repeatability across environments. Alternatively, diet may affect repeatability only when it influences the among- and within-individual variances in different way (e.g. increases V_I_ but decreases V_R,_ as in Ref. [14]). Overall the most plausible explanation is that, while we had sufficient power to detect nonzero repeatability within treatment groups, we simultaneously lacked sufficient statistical power to detect significant effects of the nutritional environment on repeatability.

### Sex-specific diet effects

If males and females prefer different nutritional environments, we may expect adaptive sex-differences in the effects of diet. In our study, we found that females consumed significantly more protein but less carbohydrate than males when given the choice (i.e., we found evidence for sex-specific nutritional preferences, Fig 3b). However, in contrast to our prediction, there were no sex-specific effects of diet on the mean level of behavioural traits. Females tend to have high protein needs because proteins are required for egg production [39]. Females with access to much protein during adulthood were thus able to invest more resources (e.g., protein) in egg production, which resulted in a sharp increase in their body weight. This may explain why differences in body weight (and its associated within-individual variance) were larger between adult diet treatments in females compared to males. In addition, diet effects on individual differentiation and behavioural plasticity were relatively stronger for male compared to female behaviour (Table 3). In our recent study, males were more vulnerable to a deficit of protein than females (Han & Dingemanse: Protein deficiency decreases male survival and the intensity of sexual antagonism, submitted), suggesting that males could be more sensitive to nutritional stress than females. If this is the case, nutritional stress is likely to affect the amount of individual differentiation and behavioural plasticity more dramatically in males. However, sex-specific effects on variance components might also be trait-specific. Therefore further investigation of the relationship between sex differences in nutritional preference and sex-specific diet effects on variance components is required.

## Conclusions

Animals often experience changes in nutritional environments, and their nutritional requirements also change throughout their lives [40]. This study demonstrates the importance of considering nutritional history as a proximate explanation for variation in the extent of individual differentiation and behavioural stability, thereby deepening our understanding of the role of ecological factors shaping these forms of variation. Furthermore, sex- and trait-specificity in effects of nutritional history imply that a fruitful line of future research would focus on the genetic architecture of dietary preferences across sexes, which may be achieved by using multivariate analyses and quantitative genetics analyses [95]. Finally, the integration of the concepts of nutritional ecology and animal personality will provide a major step towards a general understanding of behavioural evolution in changing environments [60].

## Additional file

Additional file 1: Supplementary material. **Figure S1**. Schematic representations of hypothesized population-level average behavioural responses and associated among- and within-individual variances across environments. **Figure S2**. The experimental set-up. **Figure S3**. The effect of diet and sex on the expression of behavioural and morphological traits. **Table S1**. Diet effects on variance components and repeatability of phenotypes. **Table S2**. Variance components (with standard errors in parentheses) for each unique combination of treatment for sex, and a range of phenotypic traits. **Table S3**. Additional linear mixed models to test the effect of social partner treatment on aggression and mating activity. (DOCX 479 kb)
