# Supplementary material for: You are what you eat: diet shapes body composition, personality and behavioural stability
Source: BMC Evol Biol. 2017 Jan 10;17:8. doi: 10.1186/s12862-016-0852-4 (PMC5223362; doi:10.1186/s12862-016-0852-4)
Supplement: Supplementary file 1 — Supplementary material. Figure S1. Schematic representations of hypothesized population-level average behavioural responses and associated among- and within-individual variances across environments. Figure S2. The experimental set-up. Figure S3. The effect of diet and sex on the expression of behavioural and morphological traits. Table S1. Diet effects on variance components and repeatability of phenotypes. Table S2. Variance components (with standard errors in parentheses) for each unique combination of treatment for sex, and a range of phenotypic traits. Table S3. Additional linear mixed models to test the effect of social partner treatment on aggression and mating activity. (DOCX 479 kb) [file 12862_2016_852_MOESM1_ESM.docx]

**Supplementary material**

**You are what you eat: diet shapes personality and behavioural stability**

Chang S. Han^a,b^* and Niels J. Dingemanse^a^

^a^ Behavioural Ecology, Department of Biology, Ludwig-Maximilians University of Munich, Planegg-Martinsried, Germany

^b^ Current address: School of Biological Sciences, University of Queensland, St Lucia 4072, Australia

^*^ Corresponding author: [hcspol@gmail.com](mailto:hcspol@gmail.com)


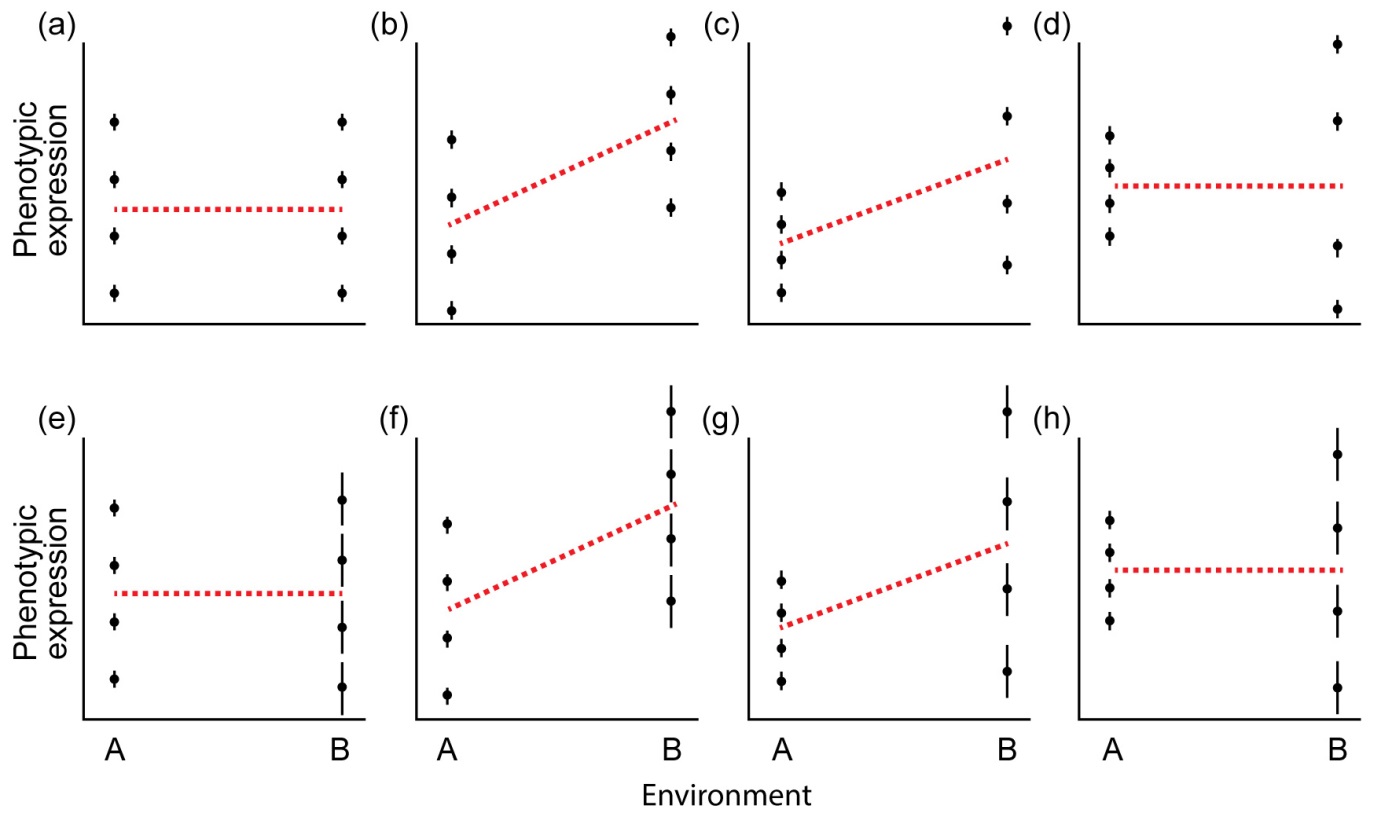


Figure S1. Schematic representations of hypothesized population-level average behavioural responses and associated among- and within-individual variances across environments. In each panel, each black line and dot represents one theoretical individual. The vertical black line represents how much each expression of an individual deviates from its individual mean (i.e., within-individual variance). The change in the length of black lines indicates the change in within-individual variance across environments. The variation among dots represents the variation among individuals in their average behaviour (i.e., among-individual variance). The red dashed line indicates the population-level mean value. The panels are examples of scenarios where the population average behaviour and its variance components change across environments. The population-level average behaviour and its variance components (both among- and within-individual variance components) can respond in the same way to the environment (a,c,f,g). Alternatively, both can independently respond to the environment (b,d,e,h). Both variance components, or one of them, can change across environments while the population average behaviour does not (d,e,h) while the reverse might also be true (b).

**
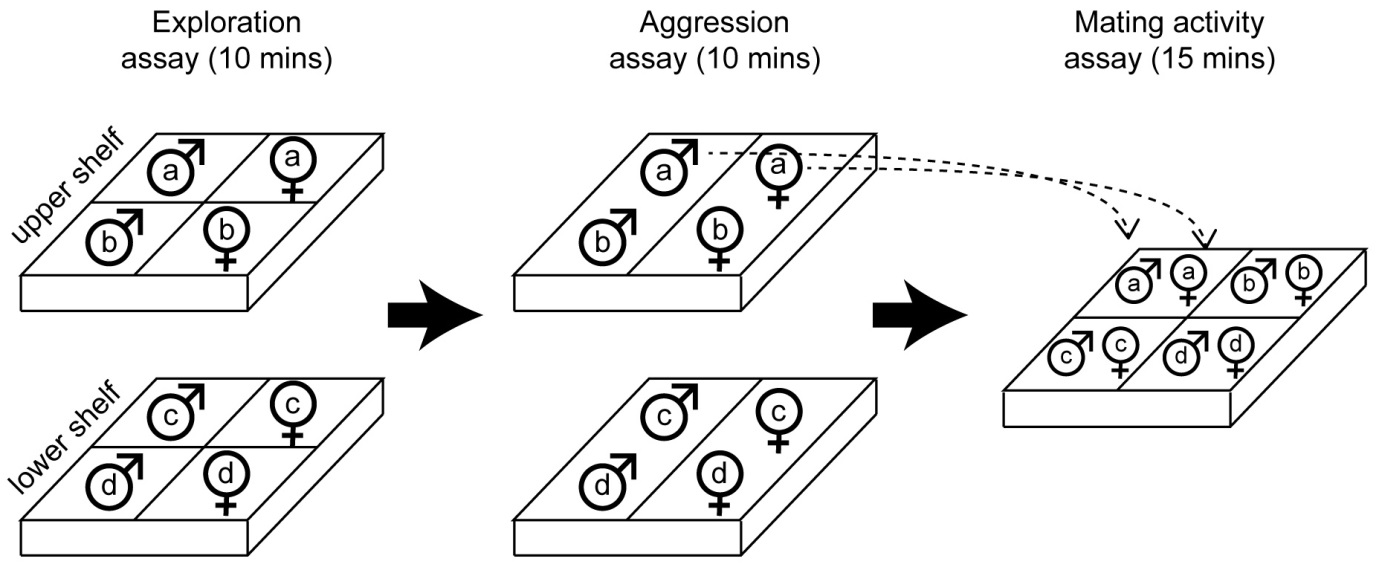
**

Figure S2. The experimental set-up. Exploration, aggression and mating activity were measured in a fixed order on the same day. 4 males and 4 females were tested simultaneously on a rack fitted with 2 shelves. Both sexes were separated during exploration and aggression assays, but then they came together in a mating activity assay.


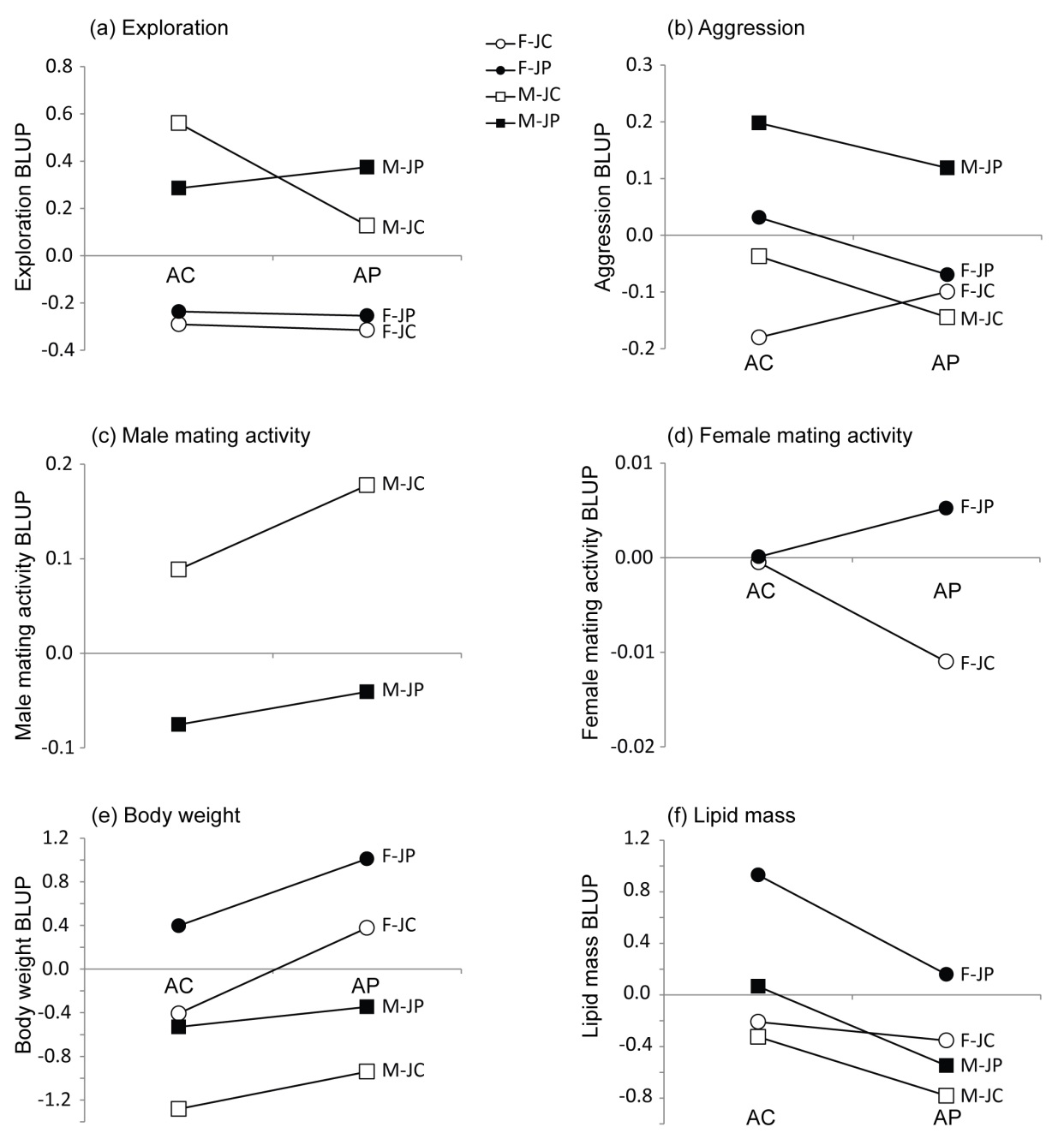


Figure S3. The effect of diet and sex on the expression of behavioural and morphological traits. The graph was based on general linear models using individual-specific BLUPs. M, male; F, female; JC, high-carbohydrate juvenile diet; JP, high-protein juvenile diet; AC, high-carbohydrate adult diet; AP, high-protein adult diet.

Table S1. Diet effects on variance components (V_I_, among-individual variance; V_R_, within-individual variance; V_P_, total variance) and repeatability (*R*) of phenotypes (EXP, exploration; AGG, aggression; MAT, mating activity; WEI, body weight; LIP, lipid mass). The numbers indicate the AIC weights of model (M) 1-4 (listed in table 1).

|  |  | Male | | | | | female | | | | |
| --- | --- | --- | --- | --- | --- | --- | --- | --- | --- | --- | --- |
|  |  | EXP | AGG | MAT | WEI | LIP | EXP | AGG | MAT | WEI | LIP |
| V_I_ | M1 (no effect)  M2 (J effect ^1^)  M3 (A effect ^2^)  M4 (combined effect ^3^) | **0.51**  0.26  0.19  0.04 | **0.54**  0.20  0.22  0.04 | 0.13  0.11  0.15  **0.61** | 0.19  **0.49**  0.19  0.13 |  | **0.47**  0.18  0.31  0.04 | **0.41**  0.28  0.26  0.05 | **0.37**  0.20  0.36  0.07 | **0.41**  0.16  0.34  0.09 |  |
| V_R_ | M1 (no effect)  M2 (J effect)  M3 (A effect)  M4 (combined effect) | **0.43**  0.30  0.20  0.07 | 0.14  **0.57**  0.06  0.23 | **0.47**  0.18  0.27  0.08 | 0.24  0.18  **0.38**  0.20 |  | **0.32**  0.28  0.14  0.27 | 0.32  **0.45**  0.14  0.09 | **0.61**  0.17  0.18  0.03 | 0.06  0.03  **0.73**  0.18 |  |
| V_P_ | M1 (no effect)  M2 (J effect)  M3 (A effect)  M4 (combined effect) | 0.25  **0.56**  0.10  0.09 | 0.04  **0.45**  0.05  **0.46** | 0.26  **0.53**  0.11  0.10 | 0.00  **0.65**  0.01  0.34 | 0.01  0.09  0.10  **0.80** | **0.48**  0.20  0.27  0.05 | 0.00  0.30  0.00  **0.70** | **0.47**  0.30  0.18  0.05 | 0.09  0.03  **0.45**  **0.43** | 0.00  0.03  0.00  **0.97** |
| *R* | M1 (no effect)  M2 (J effect)  M3 (A effect)  M4 (combined effect) | **0.56**  0.21  0.21  0.03 | **0.46**  0.32  0.17  0.05 | **0.33**  0.18  0.33  0.15 | **0.56**  0.21  0.21  0.03 |  | **0.48**  0.19  0.29  0.04 | **0.47**  0.21  0.27  0.04 | 0.33  0.16  **0.43**  0.07 | **0.56**  0.20  0.21  0.03 |  |

^1^ J effect : juvenile diet effect

^2^ A effect : adult diet effect

^3^ Combined effect: additive or non-additive effect of juvenile and adult diets

Table S2. Variance components (with standard errors in parentheses) for each unique combination of treatment for sex, and a range of phenotypic traits (a-e). CC, high-carbs juvenile & high-carbs adult diet treatment; CP, high-carbs juvenile & high-protein adult diet treatment; PC, high-protein juvenile & high-carbs adult diet treatment; PP, high-protein juvenile & high-protein adult diet treatment.

| (a) Exploration | | | | | |
| --- | --- | --- | --- | --- | --- |
| Sex | Treatment | V_R_ | V_I_ | V_partner_ | V_P_ |
| Male | CC | 27016 (5627) | 46977 (19468) | n.a. | 73992 (19808) |
| Male | CP | 20902 (4546) | 47270 (19968) | n.a. | 68172 (20217) |
| Male | PC | 31166 (4726) | 65198 (19240) | n.a. | 96364 (19529) |
| Male | PP | 28268 (3435) | 61332 (14370) | n.a. | 89600 (14563) |
| Female | CC | 14464 (2760) | 9865 (4516) | n.a. | 24330 (4849) |
| Female | CP | 18534 (3013) | 12846 (5017) | n.a. | 31380 (5429) |
| Female | PC | 15788 (1901) | 9920 (2945) | n.a. | 25708 (3222) |
| Female | PP | 11766 (1444) | 14629 (3740) | n.a. | 26395 (3875) |
|  |  |  |  |  |  |
| (b) Aggression | | | | | |
| Sex | Treatment | V_R_ | V_I_ | V_partner_ | V_P_ |
| Male | CC | 71 (110) | 109 (164) | 473 (263) | 653 (222) |
| Male | CP | 22 (23) | 487 (225) | 177 (80) | 686 (225) |
| Male | PC | 440 (214) | 490 (286) | 646 (300) | 1576 (350) |
| Male | PP | 576 (199) | 240 (158) | 308 (277) | 1124 (182) |
| Female | CC | 213 (68) | 11 (50) | 0.0001 (0) | 224 (51) |
| Female | CP | 286 (84) | 24 (62) | 0.0003 (0) | 310 (68) |
| Female | PC | 457 (155) | 22 (94) | 194 (134) | 673 (111) |
| Female | PP | 358 (73) | 106 (72) | 0.0002 (0) | 464 (73) |
|  |  |  |  |  |  |
|  |  |  |  |  |  |
| (c) Mating behaviour | | | | | |
| Sex | Treatment | V_R_ | V_I_ | V_partner_ | V_P_ |
| Male | CC | 2.83 (1.26) | 2.93 (1.63) | 1.28 (1.28) | 7.04 (1.67) |
| Male | CP | 5.57 (2.55) | 0.001 (0) | 0.15 (2.32) | 5.72 (1.11) |
| Male | PC | 4.11 (0.64) | 0.49 (0.45) | 0.001 (0) | 4.61 (0.63) |
| Male | PP | 2.78 (0.52) | 0.67 (0.38) | 1.71 (0.63) | 5.16 (0.61) |
| Female | CC | 77704 (29256) | 0.001 (0) | 14604 (26707) | 92308 (21207) |
| Female | CP | 104600 (25107) | 4955 (17982) | 0.06 (0.02) | 109555 (21111) |
| Female | PC | 94839 (15998) | 0.03 (0.006) | 30913 (16083) | 125752 (16439) |
| Female | PP | 95996 (15912) | 16960 (10517) | 5778 (10428) | 118734 (13836) |
|  |  |  |  |  |  |
| (d) Body weight ^1^ | | | | | |
| Sex | Treatment | V_R_ | V_I_ | V_partner_ | V_P_ |
| Male | CC | 5.7 (2.0) | 40.9 (16.0) | n.a. | 46.6 (16.0) |
| Male | CP | 2.3 (0.9) | 47.2 (18.3) | n.a. | 49.4 (18.3) |
| Male | PC | 7.3 (1.9) | 65.2 (18.1) | n.a. | 72.5 (18.1) |
| Male | PP | 4.7 (1.0) | 96.8 (20.9) | n.a. | 101.5 (20.9) |
| Female | CC | 9.2 (3.2) | 83.5 (28.4) | n.a. | 92.7 (28.3) |
| Female | CP | 28.0 (7.7) | 173.3 (53.1) | n.a. | 200.9 (53.1) |
| Female | PC | 13.0 (2.7) | 129.4 (28.4) | n.a. | 142.0 (28.3) |
| Female | PP | 21.0 (4.3) | 154.1 (34.7) | n.a. | 174.7 (34.7) |
|  |  |  |  |  |  |
| (e) Lipid mass ^2^ | | | | | |
| Sex | Treatment |  |  |  | V_P_ |
| Male | CC | n.a. | n.a. | n.a. | 7.7 (2.8) |
| Male | CP | n.a. | n.a. | n.a. | 3.1 (1.3) |
| Male | PC | n.a. | n.a. | n.a. | 20.0 (5.3) |
| Male | PP | n.a. | n.a. | n.a. | 9.4 (1.9) |
| Female | CC | n.a. | n.a. | n.a. | 14.0 (4.7) |
| Female | CP | n.a. | n.a. | n.a. | 8.2 (2.2) |
| Female | PC | n.a. | n.a. | n.a. | 72.0 (15.0) |
| Female | PP | n.a. | n.a. | n.a. | 25.0 (5.1) |

^1^ Variance components of body weight were multiplied by 10^4^

^2^ Total phenotypic variances of lipid mass were multiplied by 10^5^

n.a. = non-applicable, not estimated for this trait.

Table S3. Additional linear mixed models to test the effect of social partner treatment on aggression and mating activity.

|  | Aggression | Male mating  activity | Female mating  activity |
| --- | --- | --- | --- |
| *Fixed effects* |  |  |  |
| Juvenile diet (J)^a^ | **F_1,207.0_=5.36**  **P=0.02** | **F_1,96.8_=13.18**  **P<0.001** | F_1,119.9_=1.01  P=0.32 |
| Adult diet (A)^b^ | F_1,198.9_=2.80  P=0.10 | F_1,105.1_=1.42  P=0.24 | F_1,113.2_=0.06  P=0.81 |
| Sex^c^ | **F_1,185.9_=12.24**  **P<0.001** | n.a. | n.a. |
| J×A^d^ | F_1,202.8_=0.99  P=0.32 | F_1,100.4_=0.22  P=0.64 | F_1,121.1_=1.24  P=0.27 |
| J×Sex | F_1,203.6_=1.49  P=0.23 | n.a. | n.a. |
| A×Sex | F_1,198.3_=0.01  P=0.93 | n.a. | n.a. |
| J×A×Sex | F_1,200.2_=2.36  P=0.13 | n.a. | n.a. |
| Time of day | **F_1,305.8_=5.87**  **P=0.02** | **F_1,265.3_=6.05**  **P=0.02** | **F_1,290.1_=14.13**  **P<0.001** |
| Testing order | F_1,279.8_=3.59  P=0.06 | F_1,224.0_=3.64  P=0.06 | F_1,231.5_=0.07  P=0.79 |
| Shelf | F_1,371.9_=0.45  P=0.50 | n.a. | n.a. |
| Partner treatment | F_4,223.5_=0.87  P=0.49 | F_3,131.2_=0.72  P=0.55 | **F_4,123.3_=3.19**  **P=0.02** |
| Focal treatment × Partner treatment | F_10,426.2_=0.44  P=0.92 | F_9,376.0_=1.83  P=0.06 | F_9,349.1_=0.98  P=0.46 |
| Sex × Partner treatment | **F_4,230.7_=3.58**  **P=0.007** | n.a. | n.a. |
| Sex × Focal treatment× Partner treatment | F_9,426.5_=1.40  P=0.19 | n.a. | n.a. |
| Intercept | F_1,186.7_=0.54  P=0.46 | F_1,100.2_ =0.68  P=0.41 | F_1,81.5_=2.52  P=0.12 |
| *Random effects* |  |  |  |
| ID | 0.24 (0.06) | 0.15 (0.05) | 0.05 (0.05) |
| Partner ID | 0.18 (0.06) | 0.18 (0.06) | 0.15 (0.06) |

^a^ juvenile diet effect (high-carbohydrate diet as the contrast)

^b^ adult diet effect (high-carbohydrate diet as the contrast)

^c^ sex effect (females as the contrast).

^d^ interactive effect between juvenile and adult diets
